# SARS-CoV-2 infection initiates interleukin-17-enriched transcriptional response in different cells from multiple organs

**DOI:** 10.1038/s41598-021-96110-3

**Published:** 2021-08-19

**Authors:** Md Zobaer Hasan, Syful Islam, Kenichi Matsumoto, Taro Kawai

**Affiliations:** 1 Laboratory of Molecular Immunobiology, Division of Biological Science, Graduate School of Science and Technology, Nara Institute of Science and Technology (NAIST), Nara, 630-0192 Japan; 2 Research Village Kyoto, Rohto Pharmaceutical CO, Ltd, Kyoto 619-0216 Japan; 3 Laboratory of Software Engineering, Division of Information Science, Graduate School of Science and Technology, Nara Institute of Science and Technology (NAIST), Nara, 630-0192 Japan

**Keywords:** Computational biology and bioinformatics, Immunology, Infectious diseases, Inflammation, Innate immunity

## Abstract

Severe acute respiratory syndrome coronavirus 2 (SARS-CoV-2) infection has emerged as a pandemic. Paucity of information concerning the virus and therapeutic interventions have made SARS-CoV-2 infection a genuine threat to global public health. Therefore, there is a growing need for understanding the molecular mechanism of SARS-CoV-2 infection at cellular level. To address this, we undertook a systems biology approach by analyzing publicly available RNA-seq datasets of SARS-CoV-2 infection of different cells and compared with other lung pathogenic infections. Our study identified several key genes and pathways uniquely associated with SARS-CoV-2 infection. Genes such as interleukin (IL)-6, CXCL8, CCL20, CXCL1 and CXCL3 were upregulated, which in particular regulate the cytokine storm and IL-17 signaling pathway. Of note, SARS-CoV-2 infection strongly activated IL-17 signaling pathway compared with other respiratory viruses. Additionally, this transcriptomic signature was also analyzed to predict potential drug repurposing and small molecule inhibitors. In conclusion, our comprehensive data analysis identifies key molecular pathways to reveal underlying pathological etiology and potential therapeutic targets in SARS-CoV-2 infection.

## Introduction

Severe acute respiratory syndrome coronavirus-2 (SARS-CoV-2) is a novel strain of the coronavirus family and the causative viral strain of the coronavirus disease 2019 (COVID-19) pandemic. Most of the cases are acute with symptoms like fever, shortness of breath and fatigue. However, growing evidence suggests that higher mortality is associated with further longer-term health complications. Clinical manifestations of SARS-CoV-2 have been reported in which patients develop flu or pneumonia-like respiratory syndrome along with organ damage such as liver and heart^1–4^. A recent work has shown that ACE2 (host cell receptor for SARS-CoV-2 entry) expression levels do not differ based on age, sex or ethnicity^5^. This part explains the wide-spread transmission of the virus and raises the possibility of immune response as critical factor for mortality risk. Indeed, high level of pro-inflammatory cytokines were evident both in Middle East Respiratory Syndrome (MERS) coronavirus and Severe Acute Respiratory Syndrome coronavirus (SARS-CoV) infection. This results in the infiltration of immune cells, thereby promoting lung injury^6,7^. Severely ill COVID-19 patients also demonstrated higher levels of pro-inflammatory cytokines in bronchoalveolar lavage fluid (BALF) and peripheral blood mononuclear cells (PBMC)^8^. In addition, most of the COVID-19 patients exhibited an increase in inflammatory monocytes and neutrophils^9,10^.

Despite enormous effort, SARS-CoV-2 infection susceptibility index remains elusive. We have observed myriad of events including respiratory organ failure, multiple organ injury associated with cytokine-storm-mediated inflammation. Notably, about 60% of SARS-CoV infected patients had liver damage^11^ and this phenomenon was also evident in MERS-CoV infection to a lesser extent^12^. Interestingly, COVID-19 patients have also showed similar trend of liver damage associated with abnormal liver function^13^. However, whether this liver damage is due to viral infection itself or associated with the drugs used for treatment, still needs to be elucidated. Reportedly, cardiac injury has been observed in COVID-19 patients^14,15^ and a meta-analysis study also supports the notion of multiple organ damage due to SARS-CoV-2 infection.

Understanding the hyperinflammation and type of immune response is of utmost importance to design an effective therapy. The innate immune system mounts the first line of defense against viral infection at an early stage. Innate sensing of viral material initiates antiviral response by producing type I interferon (IFN) through interferon regulatory factors (IRFs) and elicits a pro-inflammatory cytokine response via NF-κB-dependent pathways^16^. Reportedly, monocytes from elderly subjects showed diminished IFNα/β level while producing pro-inflammatory cytokines upon stimulation^17^. This suggests that aging specifically impairs the IFN activation but not pro-inflammatory cytokine production. Indeed, COVID-19 and SARS-CoV patients exhibited hypercytokinemia^18,19^ with an aberrant IFN response. Hence, a more comprehensive and concerted study involving different in vitro or in vivo models are required to understand the interplay between virus and host innate immune response.

Here, we sought to characterize spatial distribution of host transcriptome in different cells upon SARS-CoV-2 infection and whether the infection generates unique and distinctive transcriptomic signature. In this regard, we have analyzed and integrated publicly available gene expression datasets. First, we have analyzed RNA-seq data from SARS-CoV-2-infected normal human bronchial epithelial (NHBE) cells, A549 cells (human lung carcinoma), primary human airway epithelial (pHAE) cultures, cardiomyocytes and liver organoids. To further understand, we have compared these data with other respiratory viruses such as SARS-CoV, MERS, Influenza A virus (IAV), and Respiratory Syncytial Virus (RSV). SARS-CoV-2 infection elicited a clearly distinctive host response in multiple cells compared with those observed in other viral infections. A computational analysis was carried out to predict potential drug candidates based on their ability to reverse SARS-CoV-2-mediated host transcriptional signature.

## Methods

### Dataset and sample information

The NCBI Gene Expression Omnibus (GEO) database (http://www.ncbi.nlm.nih.gov/geo/) has been queried using keywords “SARS-CoV-2” and “COVID-19”. The datasets were selected with stringent selection of data generated on “Homo-sapiens”. Finally, following datasets were included for this study: GSE147507^20^, GSE150392^21^, GSE153970^22^ and GSE151803^23^. Briefly, three independent biological replicates of primary human lung/airway epithelial cells or A549 cells that were mock treated or infected with SARS-CoV-2 at a Multiplicity Of Infection (MOI) of 2 (GSE147507) or 0.25 (GSE153970), for 24 h and 48 h respectively. A low MOI of 0.1 was used for 72 h in GSE150392 using iPSC derived cardiomyocytes. Liver organoid infections were performed at a MOI of 0.1 for 24 h in GSE 151,803. SARS-CoV-2 (USA-WA1/2020) was used in all these studies. In addition to SARS-CoV-2, GSE47960 and GSE100504 have been used to analyze host response to SARS-CoV and MERS viruses respectively. Primary human lung/airway epithelial cells were infected at an MOI of 2 and 5 for SARS-CoV and MERS, respectively in these studies. GSE147507 dataset was used for RSV- and IAV-infected host transcriptome. A complete list containing cell types, MOI, infection period has been prepared as supplementary table 1 (see supplementary information file).

### Data processing and analysis

Raw sequencing reads were quality controlled and aligned to the human genome (hg19) using the RNA-Seq Alignment App on Basespace (Illumina, CA, USA), followed by differential expression analysis using DESeq2^24^. Differentially expressed genes (DEGs) were characterized for each sample (p adjusted-value < 0.05 and log2FC > 1). Volcano plots were constructed using custom scripts in R. A large volume of DEGs were identified with low count and highly dispersed genes, hence shrinkage estimator ‘apeglm’^25^ was used in order to remove noise and visualize the DEGs with significant differences in mock treated vs SARS-CoV-2 treated group. Particularly this statistical approach preserves true, large differences in log fold change (LFC) across conditions and is superior to common methods in ranking of genes by LFC in presence of low counts**.** In brief, apeglm utilizes heavy-tailed Cauchy distribution instead of normal distribution on the effect sizes, with fixed shape and scale adapted to the distribution of observed maximum likelihood estimates (MLE) for all genes. Then a LaPlace approximation was used to provide shrinkage estimates and corresponding standard deviation. This eliminated further needs for filtering rules or pseudo counts and maximizes the power of the current data to estimate the effect size for each gene.

### Functional enrichment analysis

A web-based tool, Metascape^26^ was used for functional enrichment of DEGs. Metascape queries publicly available databases, e.g. Gene Ontology, Kyoto Encyclopedia of Genes and Genomes (KEGG)^27^ and assigns DEGs to their respective enriched pathways by calculating the pairwise similarity between any two terms. Hypergeometric test and Benjamini–Hochberg p-value correction algorithm have been used to identify statistically significant enriched ontology terms. The Bioconductor cluster Profiler package v3.14.3^28^ and STRING v11^29^ have also been used to develop gene to pathway and protein–protein interaction (PPI) networks of upregulated DEGs and Cytoscape application v3.7.1^30^ was used for plotting genes based on the count and p-value.

### Disease similarities and drug predictive analysis

DisGeNET database^31^ was employed to identify disease similarities with enriched DEGs. A minimum count of 3 genes, with a p-value < 0.01 and an enrichment factor > 1.5 (the enrichment factor is the ratio between the observed counts and the counts expected by chance) were collected and grouped into clusters based on their membership similarities. Accumulative hypergeometric distribution was employed for calculating p-value. The L1000FDW web tool^32^ was utilized for potential drug candidate search for the treatment of SARS-CoV-2 infection. L1000FWD computes similarity between a given set of genes with the Library of Integrated Network-based Cellular Signatures (LINCS)-L1000 data and predicts compounds or drugs that reverse the input transcriptomic signature.

## Results

### DEGs of SARS-CoV-2 infection

**Figure 1:**
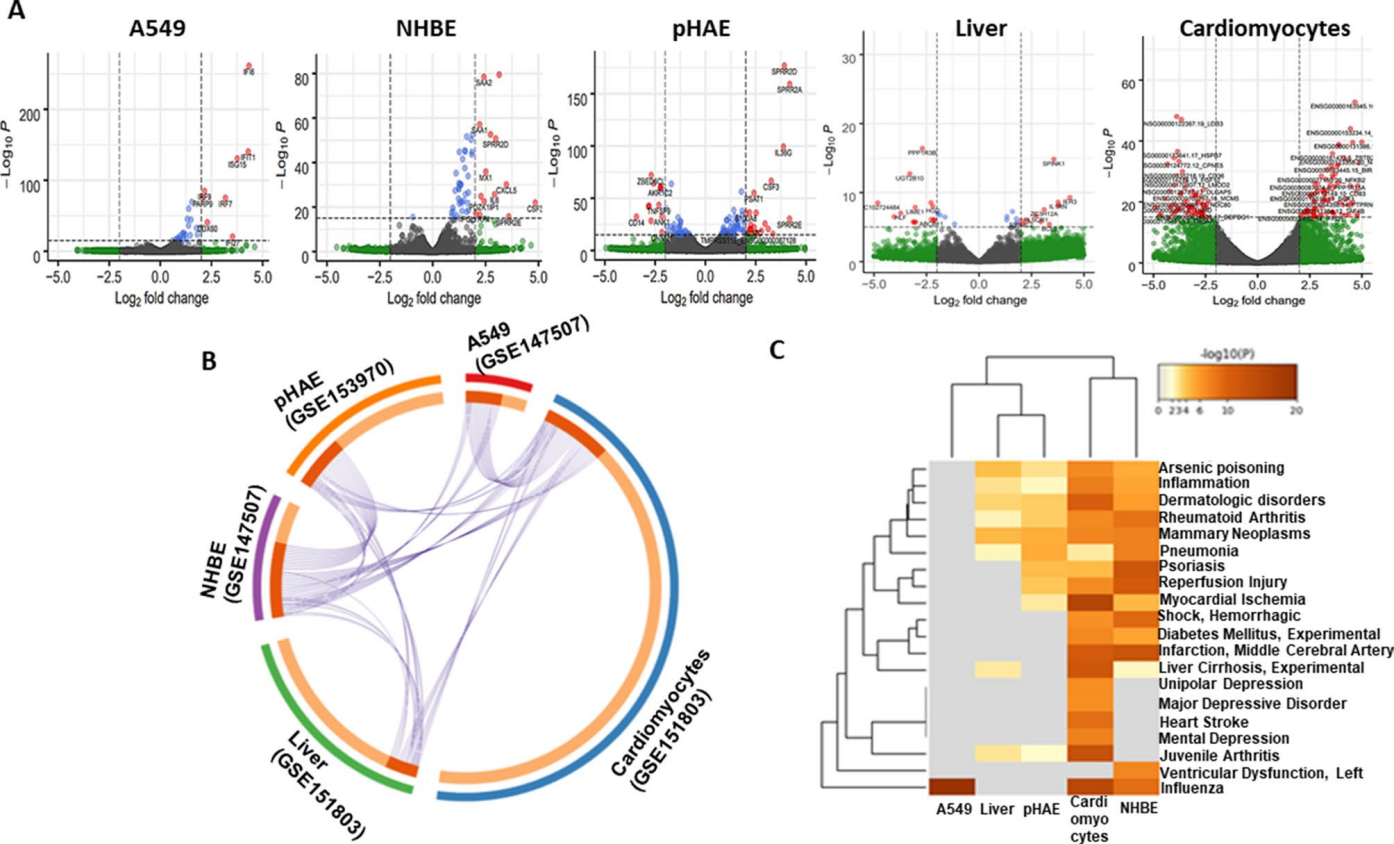
DEGs of SARS-CoV-2 infection. (**A**) Volcano plots indicating DEGs of A549, NHBE, pHAE, Liver organoids and Cardiomyocytes upon SARS-CoV-2 infection for 24 h. DEGs (p-adjusted < 0.05) with a log2FC of more than 2 are indicated in red. Non-significant DEGs with a log2FC of more than 2 are indicated in green. (**B**) Circos plot showing overlapping among the genes significantly upregulated following SARS-CoV-2 infection using purple lines. (**C**) Hierarchical clustering of the top most enriched diseases by the upregulated genes from different datasets. Dendogram is colored by the p values where grey cells indicate the lack of significant enrichment.

In order to determine and compare host cell response against SARS-CoV-2 infection, we first analyzed various publicly available RNA-sequencing datasets. We identified numerous DEGs in lung, liver and heart cells (Fig. 1A). Due to large number of DEGs, we employed more stringent parameter and used ‘apeglm’ algorithm in Deseq2 program to identify highly differential genes. This significantly narrowed down the gene list and interestingly most of the DEGs belong to an upregulated category (Fig. 1A). We could not find any significant association among those few downregulated genes. For subsequent analysis, mostly upregulated genes have been used. Notably, chemokine genes (CXCL1, CXCL3, CXCL5, CXCL8, CCL20) and ISGs (IFITM1, IFI44L, IFI27) dominated this list (See supplementary file 1 for the gene lists).

An MA plot showing A549 cells had very few DEGs compared with other cells used in the study (See Figure S1). However, supplementation with putative SARS-CoV-2 receptor ACE2 in A549 cells resulted in higher number of modulated transcriptome (see Figure S2A). A circos plot showing association of DEGs among various datasets. Particularly, NHBE cells share many DEGs with pHAE cells (Fig. 1B). Then, the DEGs were screened for their approximate association in disease-gene networks using DisGeNET database. Strikingly, enrichment of our upregulated genes could link most of the clinical features such as pneumonia, influenza, myocardial ischemia, hemorrhagic shock, etc. observed in COVID-19 patients. (Fig. 1C).

### Functional annotation and pathway enrichment of DEGs

**Figure 2:**
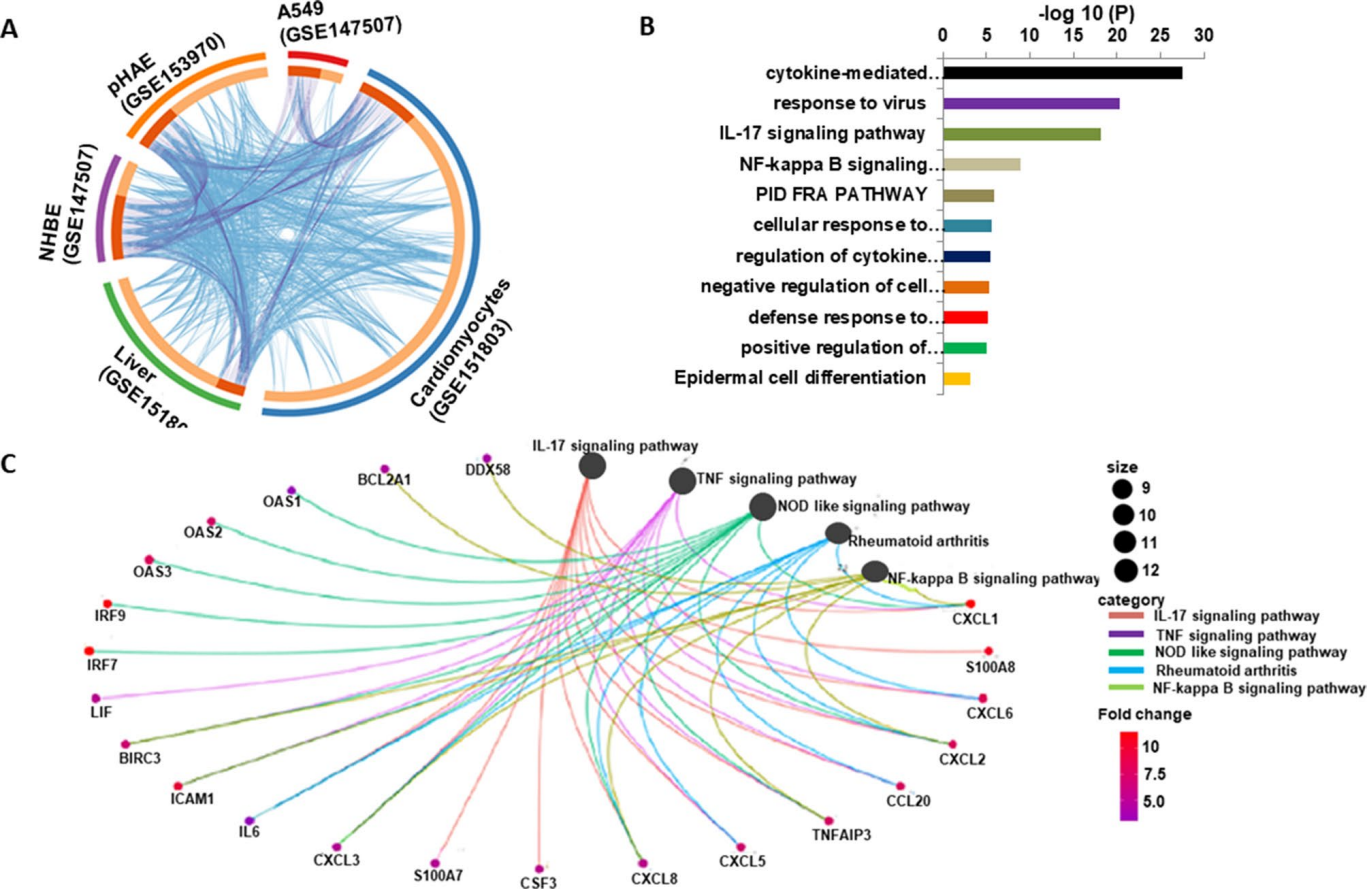
SARS-CoV-2 infection induces inflammatory signaling. (**A**) Circos plot showing the overlapping among genes significantly upregulated upon SARS-CoV-2 infection of different cells. Purple lines represent shared genes by various cells. Blue lines represent the different genes that fall in the same ontology term. (**B**) Hierarchical clustering of the top most enriched KEGG pathways from different datasets. Dendogram is colored by the p values where grey cells indicate the lack of significant enrichment. (C) Circos plot showing collated shared genes from different datasets, genes are assigned to their designated KEGG pathways.

Next, we performed gene set functional enrichment analysis using KEGG pathway. Circos plot showing many distinct genes in one dataset are ontologically connected to the ontological features in another dataset (Fig. 2A). Key pathways shared by genes from most of the datasets were cytokine-cytokine receptor signaling, IL-17 signaling pathway, NOD-like receptor-mediated signaling, and Measles (Fig. 2B). Interestingly, very few genes were upregulated in A549 cells and were not enriched into IL-17 signaling pathway. We then analyzed transcriptome of A549 cells supplemented with putative SARS-CoV-2 receptor, ACE2. ACE2 receptor supplementation induced expression of transcripts that were shared with other cells (See Figure S2A) and enriched in IL-17-mediated signaling pathways (See Figure S2B).

DEGs were then ranked based on their frequency of distribution in datasets and number of associated pathways. We selected top 50 upregulated genes that were present in at least two different cells. Pathway enrichment analysis based on gene count assigned in KEGG pathway for upregulated DEGs revealed IL-17 signaling, NOD-like receptor-mediated signaling, and the TNF signaling (Fig. 2C) amongst the most regulated pathways by these genes. Among other statistically significant enriched terms, pathways related to NF-$\kappa$B and rheumatoid arthritis were also evident (Fig. 2C). In addition, analyzed data from BALF collected from COVID-19 patients in China (Xiong et al. 2020) followed a similar trend of enrichment identified from our analysis (See Figure S3). However, we did not observe strong similarities with our host transcriptome data when PBMC transcripts were used for analysis from the same study group in China.

### SARS-CoV-2 transcriptomes are enriched into IL-17 pathway

**Figure 3:**
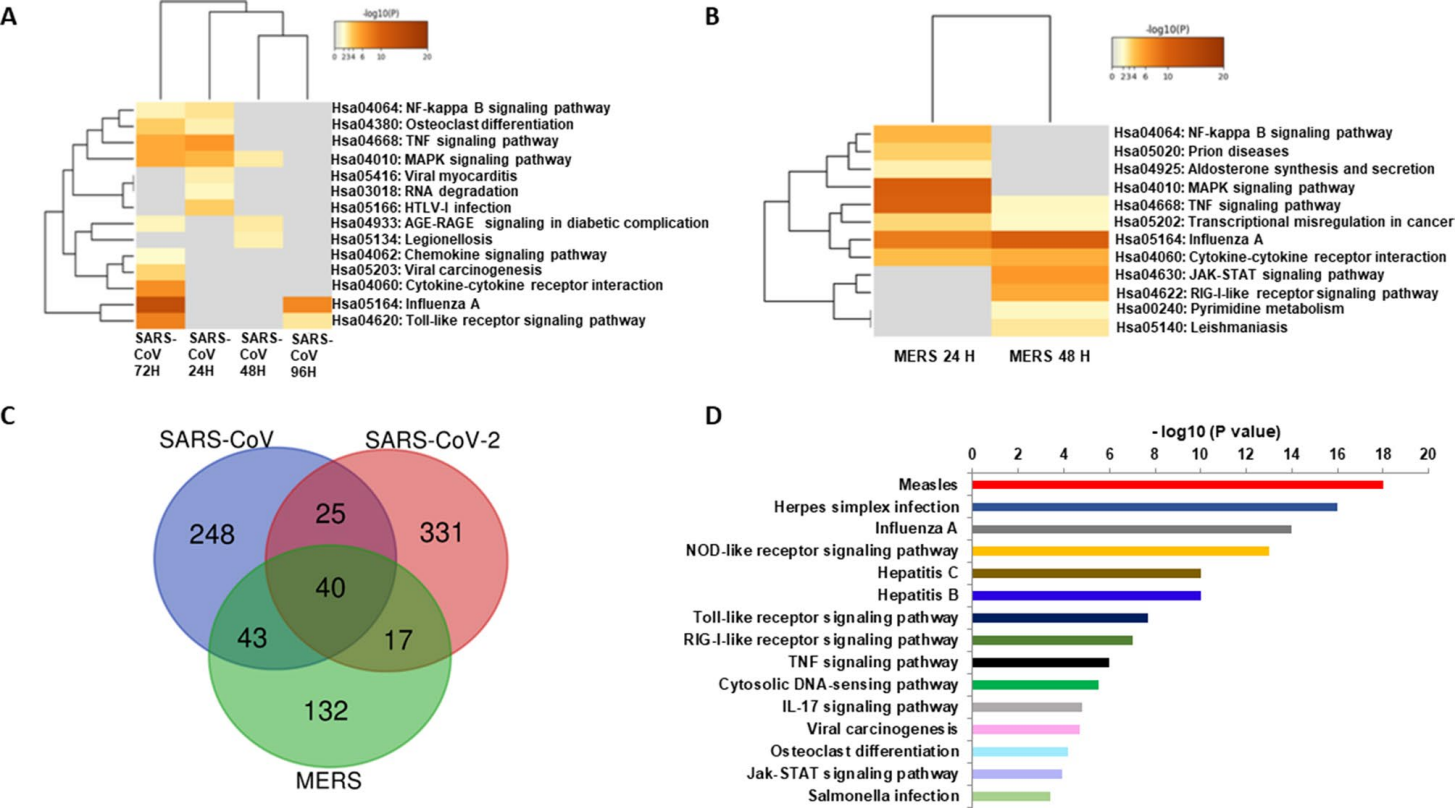
SARS-CoV, SARS-CoV-2 and MERS-infected transcriptome activates innate viral sensing pathways. (**A**) Hierarchical clustering of the top most enriched terms in KEGG pathway by genes significantly upregulated at different time points (24 – 96 h) upon SARS-CoV infection. (**B**) Hierarchical clustering of the top most enriched terms in KEGG pathway by genes significantly upregulated at different time points (24 – 48 h) upon MERS infection. Dendogram is colored by the p values, and grey cells indicate the lack of significant enrichment. (**C**) Shared DEGs in SARS-CoV, SARS-CoV-2 and MERS infection. Venn diagram depicting genes shared and/or unique between each comparison. (**D**) Barplot showing most enriched KEGG pathways upon collation of 40 shared DEGs from SARS-CoV, SARS-CoV-2 and MERS infection. Y-axis represents KEGG pathway and X-axis represents p-value (-log). Higher –log P indicates smaller and more significant p-value.

To further our understanding of the SARS-CoV-2-driven IL-17-enriched transcriptome, we have analyzed microarray data from 24 to 96 h and 24–48 h for SARS-CoV and MERS infection, respectively. SARS-CoV and MERS upregulated genes mainly enriched into MAPK, NF-$\kappa$B and TNF-α signaling pathways (Fig. 3A,B). Next, we compared genes that were upregulated in SARS-CoV, SARS-CoV-2- and MERS-infected cells (Fig. 3C). Enrichment of 40 shared genes showed ‘Measles’, ‘NOD-like receptor signaling’, ‘TLR signaling’, ‘RLR signaling’, and ‘TNF signaling’ (Fig. 3D). IL-17 signaling was also among the enriched pathway, however it was not as strongly activated as we observed in all the datasets of SARS-CoV-2 infection.

**Figure 4:**
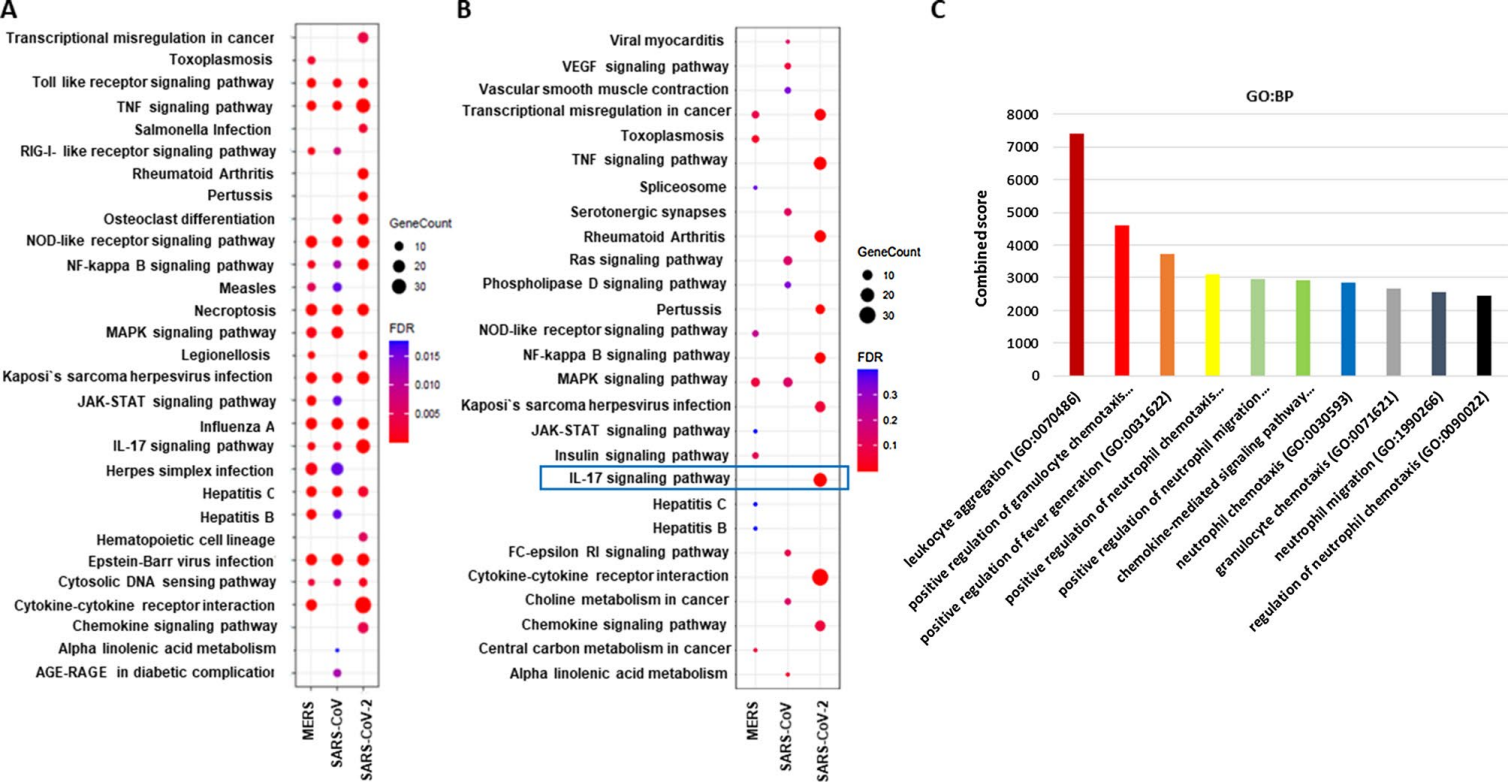
SARS-CoV-2 unique genes trigger IL-17 signaling pathway. (**A**) Dotplot visualization of enriched KEGG terms in SARS-CoV, SARS-CoV-2 and MERS infection showing all upregulated genes. Gene enrichment analyses were performed using Network analyst against the KEGG pathway. (**B**) Dotplot visualization of enriched KEGG terms in SARS-CoV, SARS-CoV-2 and MERS infection of uniquely upregulated genes in each infection. Gene enrichment analyses were performed using Network analyst against the KEGG pathway. The color of the dots represents the false discovery rate (FDR) value for each enriched KEGG term, and size represents the number of hit genes found in the datasets. (**C**) Barplot showing gene ontology for enriched biological process from genes upregulated in IL-17 pathway upon SARS-CoV-2 infection only. Biological processes were ranked based on combined score from the P value calculated by Fisher exact test and multiplying that with Z-score.

Next, we performed a gene set enrichment of all uniquely upregulated DEGs for each of the infections. We have observed during time course infection of SARS-CoV and MERS, grouping all DEGs from different time points enriched into TLR and RLR signaling pathways, along with the NF-$\kappa$B and MAPK pathways. Of note, SARS-CoV-2-infected transcripts did not trigger RLR signaling while induced strong cytokine responses mediated by NF-$\kappa$B signaling (Fig. 4A). On the other hand, SARS-CoV-2-infected unique transcriptomes predominantly enriched into ‘IL-17 signaling pathway’ while both SARS-CoV and MERS-infected unique transcripts did not. This data indicates that SARS-CoV-2 infection induces a plethora of chemokines that mediates a strong inflammatory response driven by IL-17 signaling (Fig. 4B).

We then sought to relate these unique IL-17 specific transcripts to the biological processes. Notably, ‘leukocyte aggregation’, ‘positive regulation of granulocytes/neutrophils’, and ‘fever generation’ were the top biological processes modulated by these transcripts (Fig. 4C). This demonstrated striking similarities between phenotypes observed in COVID-19 patients and biological processes controlled by the IL-17 specific transcripts.

### Comparative analysis with other respiratory virus infections

**Figure 5:**
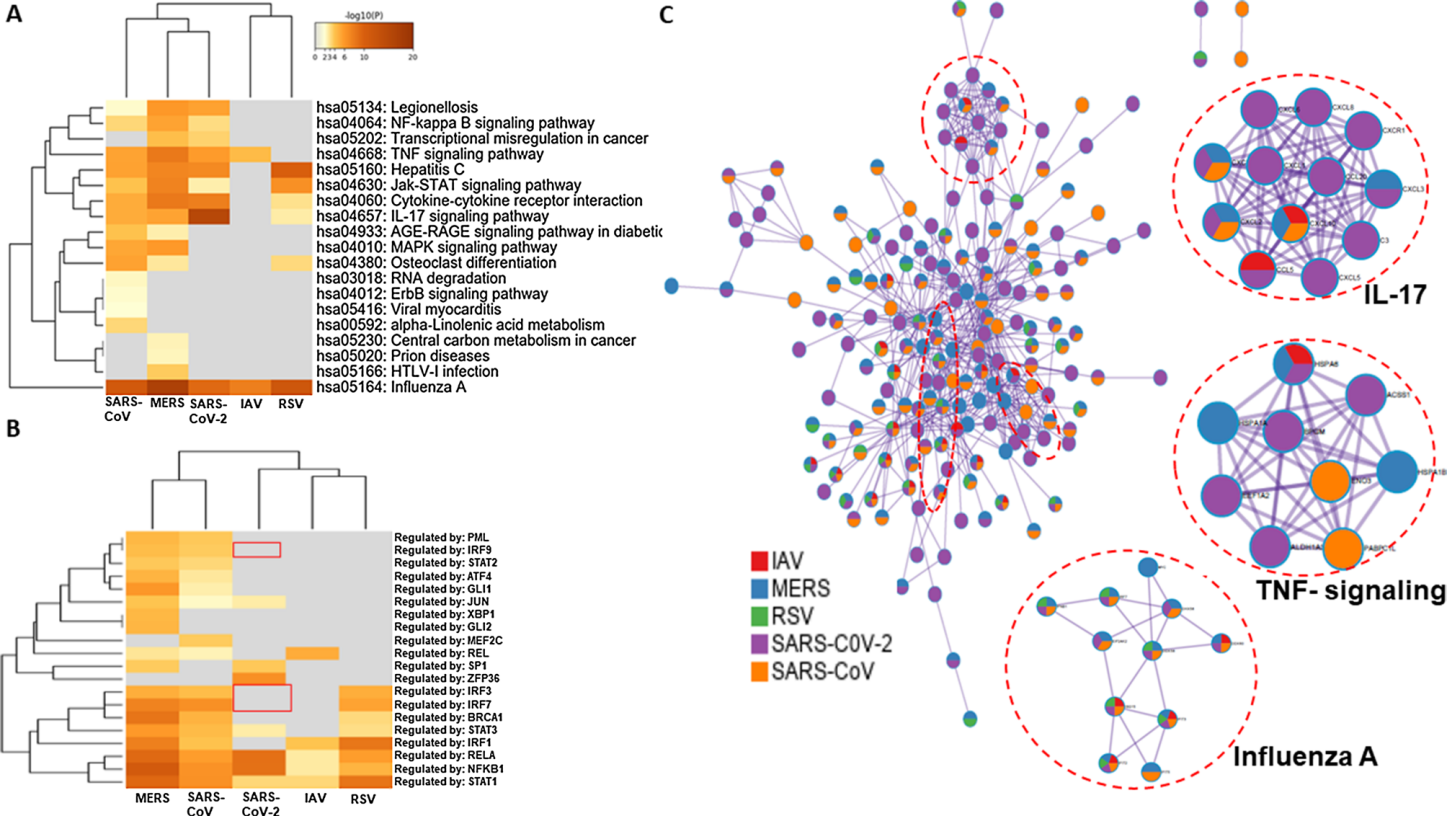
SARS-CoV-2 distinctively activates NF-$\large \kappa{\mathrm{\rm}}$B- but not IRF-mediated signaling. (**A**) Hierarchical clustering of the top most enriched terms in KEGG pathway by genes significantly upregulated upon SARS-CoV, SARS-CoV-2, MERS, RSV and IAV infection. (**B**) Hierarchical clustering of the top most enriched transcription factors for significantly upregulated genes using TRRUST database. Dendogram is colored by the p values, and grey cells indicate the lack of significant enrichment. (**C**) Enrichment network visualization for results from all datasets, where nodes are represented by pie charts with color codes depicting their designated datasets.

We extended our analysis further by including transcriptional data from respiratory syncytial virus (RSV) and Influenza A Virus (IAV). Hierarchical clustering of transcriptional responses positioned SARS-CoV-2 along with other coronaviruses while RSV and IAV belong to the same cluster (Fig. 5A). Further analysis showed that SARS-CoV-2 transcriptional regulation was mainly controlled by NF-$\kappa$B (RELA) while IRF mediated signaling was not activated. On the contrary, both SARS-CoV and MERS mediated transcriptional responses triggered IRF mediated signaling cascade (Fig. 5B).

Next, we developed protein–protein interaction (PPI) networks with the upregulated DEGs in respiratory infections using cytoscape. KEGG categories “IL-17 signaling”, “Influenza A signaling” and “TNF signaling” formed most densely connected subnetworks, which are mainly composed of ribosomal proteins and chemokines, respectively. Most of the genes in IL-17 signaling were representative of SARS-CoV-2-infected transcriptome as denoted by color (Fig. 5C). On the other hand, Influenza A signaling pathway was shared by most of the respiratory viruses and possibly produce similar phenotypes observed in those respiratory illness mediated by viral infection.

### Potential therapeutic intervention based on transcriptome signature

**Figure 6:**
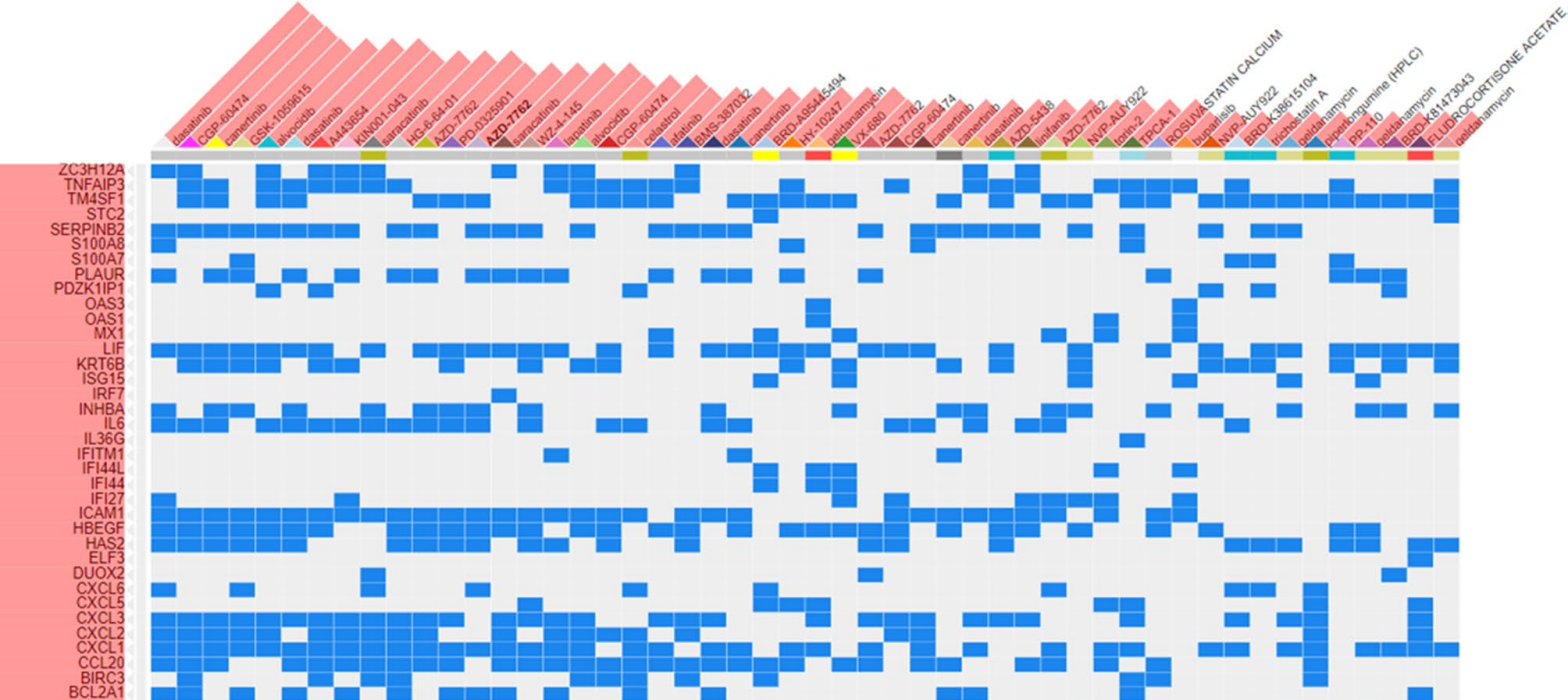
SARS-CoV-2 transcriptome-based drug prediction. L1000CDS2 visualization of drug-induced signature. Significantly upregulated top 50 genes found in at least two datasets were used as input dataset. Blue square represents drug that may inhibit gene(s).

Reverse signature perturbation analysis was performed using the upregulated DEGs identified upon SARS-CoV-2 infection (Fig. 6). Potential drugs have been clustered according to their ability to reverse the upregulated DEGs using the web-based tool L1000CDS2. Among the identified drugs; Saracatinib showed in vitro inhibition of MERS replication^33^. Besides, Dasatinib and Imatinib from our list have also been reported to inhibit SARS-CoV and MERS infection in Vero E6 cells^34^. This suggests that the repurposing of drugs based on host transcriptome may be an effective strategy until a safe and effective vaccine is developed.

## Discussion

Viruses utilize host cell machinery and modulate host cell transcriptome either for their replication or for evading host immune responses. Hence, transcriptional profiling of host cell would help us realize changes in the genetic landscape during viral infection. In this regard, we have analyzed differentially expressed genes in SARS-CoV-2-mediated infection using various in vitro cellular systems. Datasets used in our study are mostly obtained using various cells from lungs along with cardiomyocytes and liver organoids. Despite being a respiratory virus, SARS-CoV-2 has been reported to impair liver function^35^ and has active transcription site in cardiomyocytes of deceased patients^36^. Notably, ACE2 expression is high in cardiomyocytes^37^ and in general the expression is relatively higher in liver and heart when compare with lungs^38^. Thus, it is important to carry out a comparative transcriptional response in cells from different organs.

We observed a consistent enrichment of cytokine response in all the datasets used in this meta-analysis. Notably, monocyte or neutrophil recruiting chemokines such as CCL20, CXCL1, CXCL3 and CXCL8 have been upregulated. This data is in sync with the elevated level of circulating neutrophils observed in COVID-19 patients along with a decrease in lymphocytes^39–41^.

Pattern recognition receptors such as Toll-like receptors (TLRs) and RIG-I like receptors (RLRs) sense viral single or double-stranded RNA to produce inflammatory cytokines and type I IFN^42^. We observed that IRF7 and IRF9 were upregulated in SARS-CoV-2 infection. This in turn may stimulate production of interferon stimulated genes (ISGs) to produce an antiviral state. Indeed, several ISGs such as IFI1-3, IFI27, IFITM3, OAS1 and OAS3 have been upregulated. This possibly indicates initial host cellular response in order to inhibit viral replication. However, severe viral load may overwhelm type I IFN response and dictate outcome of infection. Of note, SARS-CoV and MERS-CoV infection exhibited similar outcome depending on the timing of type I IFN production. A delayed type I IFN production results in higher viral replication, cellular damage and production of cytokine storm, whereas an earlier production of type I IFN could protect from lethal effect^43,44^.

COVID-19 patients with severe respiratory symptoms had higher levels of Tumor necrosis factor-α (TNF-α) and IL-6^45^. It is worth mentioning that IL-6 was not only expressed in SARS-CoV-2-infected cells but was also involved with most of the enriched pathways in our analysis. A recent study^45,46^ showed effective inhibition of cytokine storm by blocking IL-6 with monoclonal antibody Tocilizumab, supporting the notion that IL-6- mediated inflammation contributes to the disease severity. Both IL-6 and IL-17 have been reported with protecting virally infected cells from apoptosis and thereby promoting viral persistence^47^.

All the SARS-CoV-2 datasets used in this study showed particularly strong inflammatory response triggered with IL-17 activation. Previous studies observed elevated IL-17 expression was conjoined with impaired IRF7 upregulation and IFNα responses in herpes simplex virus 2 infection of aged mice. Indeed, IL-17 inhibition could prevent death of aged mice during viral infection^48,49^. Our data analysis indicated the importance of IL-17 in SARS-CoV-2 infection. Meanwhile IL-17 has also been reported to be increased in critically ill patients compared with non-severe patients^3^. However, whether this upregulation is the direct effect of SARS-CoV-2 infection or not remains unclear. A very recent study published in April 2021 showed that SARS-CoV-2 open reading frame 8 (ORF8) binds to IL17 receptor and activates IL17 signaling pathway. Blocking IL17RA with an antibody reduced IL17 mediated inflammation in lung and liver in SARS-CoV2 ORF8 pseudovirus infected mice^50^. Indeed, a 382-nucleotide deletion variant of SARS-CoV-2 with abolished ORF8 expression was reported with milder symptoms in hospitalized patients in Taiwan and Singapore^51,52^. This report clearly supports our finding on SARS-CoV-2 distinctively initiates IL17 mediated inflammatory response and may aggravate disease severity. Of note, pro-inflammatory cytokine storms have already been reported to be damaging in other organs of COVID-19 patients^53^. Hence, blocking IL-17 could be a viable strategy to reduce multiple organ damage and disease severity.

It is still obscure how viruses induce a proinflammatory response whilst keeping immune homeostasis at bay. SARS-CoV-2 or H1N1 2009 infections showed higher IL-1RA and IL-6 levels along with very low viral load in the lung in severely ill people^54^. This raises an interesting possibility as to whether viruses blindside the innate immune system by maintaining a low amplification or self-replication state while causing local inflammation or tissue damage. Our comparative transcriptional analysis of SARS-CoV-2 and other viruses supports this notion. As evident from our observation, NF-κB is the most significant transcription factor that modulates host transcriptional responses upon SARS-CoV-2 infection. Despite producing ISGs, an absence of IRF3 or IRF7 regulated transcriptional processes was notable in SARS-CoV-2 infection. On the other hand, SARS-CoV and MERS transcriptomics were at least partly regulated by IRF1, IRF3 and IRF7. Of note, a recent work showed that SARS-CoV-2 proteases could cleave IRF3 directly and resulted in blunted IFN production^55^. This accumulating evidence suggests that SARS-CoV-2 launches a pro-inflammatory response while specifically blocking antiviral responses.

Despite showing a variable clinical spectrum, severe COVID-19 patients commonly exhibited shortness of breath, and production of pro-inflammatory cytokines such as IL-6, IL-8, IL-1β, IL-1RA^3,56^. Classic clinical symptoms predominantly indicate lung associated phenotypes, however recent evidence indicated that mortality rate is higher in cardiac patients^57,58^. Our gene set enrichment also showed that SARS-CoV-2 infected cardiomyocytes have higher expression of genes that may induce myocardial ischemia and cerebral arterial infarction.

A consistent observation in each of our SARS-CoV-2 datasets was the presence of chemokines and inflammatory cytokines. Hence, we used a reverse transcriptome signature approach for predicting drugs that are approved by FDA or in clinical trials. This analysis predicted that Dasatinib, CGP-60474, Canertinib, Alvocidib, Saracatinib etc. can perturb and reverse host transcriptional signature of SARS-CoV-2 infection. Reportedly, CGP-60474 could inhibit IL-6 and TNFα to alleviate LPS-induced sepsis in mice^59^.

We have observed distinct transcriptional responses mediated by SARS-CoV-2 in cell culture-based studies. However, MERS and IAV datasets used in our study have different MOI than that of SARS-CoV and SARS-CoV-2. MERS have higher MOI but similar infection period of 24–48 h than two other coronaviruses used in the analysis. On the other hand, IAV infection was done at slightly higher MOI than SARS-CoV or SARS-CoV-2. Interestingly, MERS infection showed late viral peak compare with SARS-CoV and SARS-CoV-2^60–62^, whilst IAV showed faster inflammatory and antiviral responses than SARS-CoV-2^63^. This clearly explains the reasoning of using slightly higher MOI for MERS and shorter infection period with IAV.

Despite comprehensive analysis of the publicly available datasets, there may be some limitations as well. As the percentage of lethality is higher in aged population, future studies using aged or senescent cells would clarify the discrepancies between young and aged in terms of infection and mortality rate. In addition, all the studies used a single cell type in the absence of immune cells, which may not reflect true intercellular communications take place in vivo. As activation of immune system varies in age groups, further research should be done using a multicellular system. As we have seen that these cellular models of infection secret numerous chemokines and particularly able to trigger immune cell activation and chemotaxis, future studies should be directed towards validating our data in moderate and severe COVID-19 patients.

**Figure 7:**
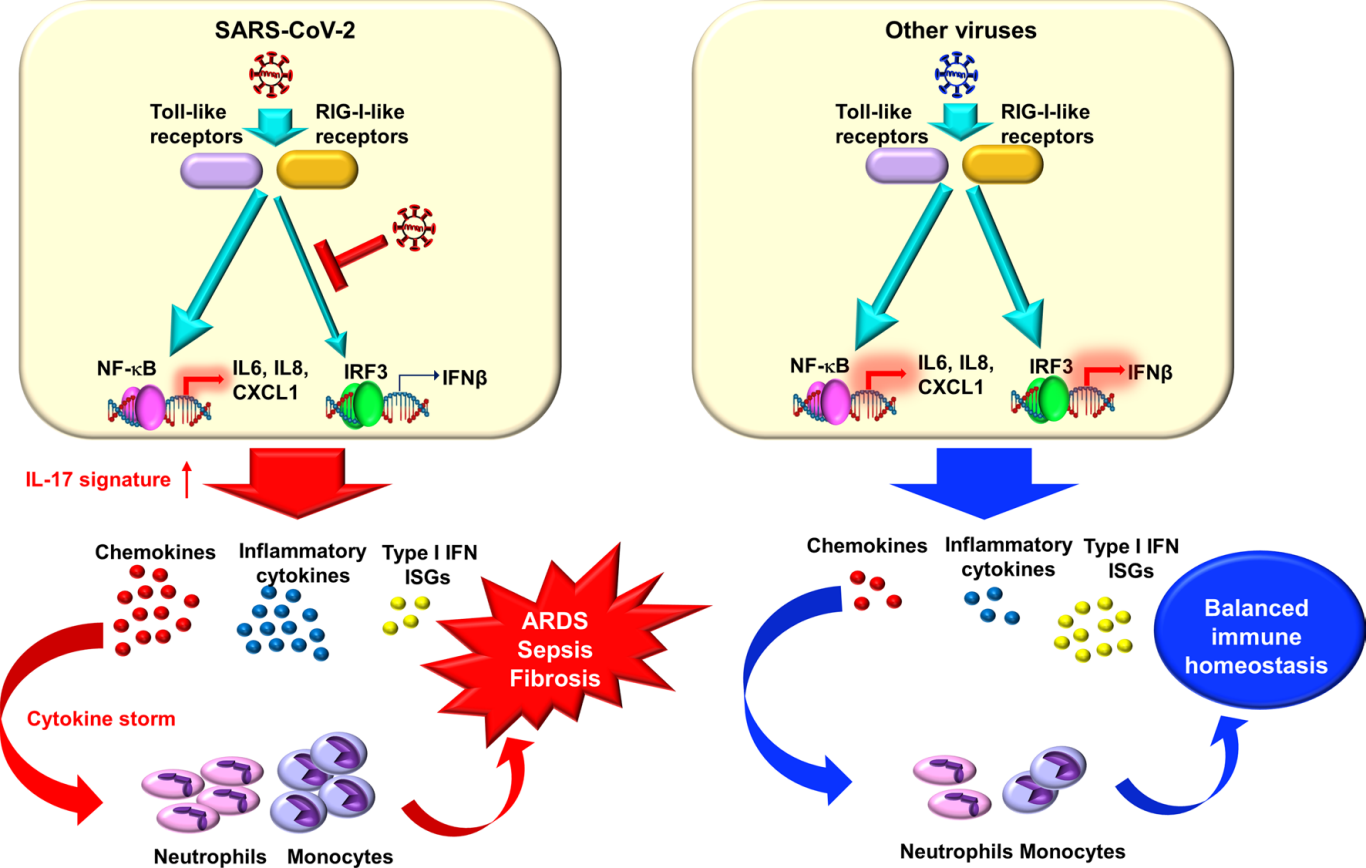
Summary of transcriptional differences among SARS-CoV-2 and other respiratory viruses. SARS-CoV-2 infection initiates a predominant IL-17 enriched chemokine transcriptional response whilst producing a low to moderate antiviral response by impairing interferon regulatory factors driven transcriptional process. This results in disproportionate immune response and recruitment of innate immune cells that ultimately leads to complications such as ARDS, sepsis or fibrosis in COVID-19 patients, On the other hand, other respiratory viruses trigger both inflammatory and antiviral transcriptional response in host cells, and thereby maintain a steady immune homeostasis.

Taken together, our comprehensive data analysis showed that SARS-CoV-2 initiated a distinct IL-17-driven inflammatory response irrespective of the cells used in various studies (Fig. 7). Overall, our data analysis showed unique and distinctive SARS-CoV-2-induced transcriptional responses compared with other respiratory viruses.

## Supplementary Information

Supplementary Information 1.

Supplementary Information 2.

## Data Availability

The datasets and code used in this study are publicly available and comprehensively described in the methods section. Any other necessary information would be provided upon reasonable request.
