# Supplementary material for: SARS-CoV-2 infection initiates interleukin-17-enriched transcriptional response in different cells from multiple organs
Source: Sci Rep. 2021 Aug 19;11:16814. doi: 10.1038/s41598-021-96110-3 (PMC8376961; doi:10.1038/s41598-021-96110-3)
Supplement: Supplementary file 1 — Supplementary Information 1. [file 41598_2021_96110_MOESM1_ESM.docx]

**SARS-CoV-2 infection initiates interleukin-17-enriched transcriptional response in different cells from multiple organs**

Md Zobaer Hasan^1^, Syful Islam^2^, Kenichi Matsumoto^2^ and Taro Kawai^1^*

^1^ Laboratory of Molecular Immunobiology, Division of Biological Science, Graduate

School of Science and Technology, Nara Institute of Science and Technology (NAIST),

Nara 630-0192, Japan.

^2^ Laboratory of Software Engineering, Division of Information Science, Graduate

School of Science and Technology, Nara Institute of Science and Technology (NAIST),

Nara 630-0192, Japan.

*To whom correspondence should be addressed

Taro Kawai (tarokawai@bs.naist.jp)

Laboratory of Molecular Immunobiology, Division of Biological Science, Graduate

School of Science and Technology, Nara Institute of Science and Technology

(NAIST), Nara 630-0192, Japan.

Tel: +81-7-4372-5550 Fax: +81-7-4372-5539

**Supplementary Figures**

| Dataset (GSE number) | Virus type | Cell type | Multiplicity of Infection (MOI) | Treatment Period |
| --- | --- | --- | --- | --- |
| GSE47960 | **SARS-CoV** | **HAE** | **2** | **24 hours**  **48 hours**  **72 hours**  **96 hours** |
| GSE100504 | **MERS-CoV** | **HAE** | **5** | **24 hours**  **48 hours** |
| GSE147507 | **SARS-CoV-2** | **A549/A549-ACE2** | **0.2-2** | **24 hours** |
| GSE147507 | **SARS-CoV-2** | **NHBE** | **2** | **24 hours** |
| GSE147507 | **IAV** | **NHBE** | **3** | **12 hours** |
| GSE147507 | **RSV** | **A549** | **2** | **24 hours** |
| GSE153970 | **SARS-CoV-2** | **A549** | **0.25** | **72 hours** |
| GSE150392 | **SARS-CoV-2** | **Cardiomyocytes** | **0.1** | **72 hours** |
| GSE151803 | **SARS-CoV-2** | **Liver organoids** | **0.1** | **24 hours** |

**Supplementary Table 1:References of the datasets used in the study.** This table summarizes the datasets collected for the comparative analysis of SARS-CoV, MERS-CoV, SARS-CoV-2, IAV and RSV. Cell type, multiplicity of infection (MOI) and infection period have been listed accordingly. HAE= Human Airway Epithelial cells, A549=Adenocarcinomic Human Alveolar Basal Epithelial cells, A549-ACE2= Adenocarcinomic Human Alveolar Basal Epithelial cells expressing human ACE2 (SARS-CoV-2 putative receptor), NHBE= Normal Human Bronchial Epithelial cells.


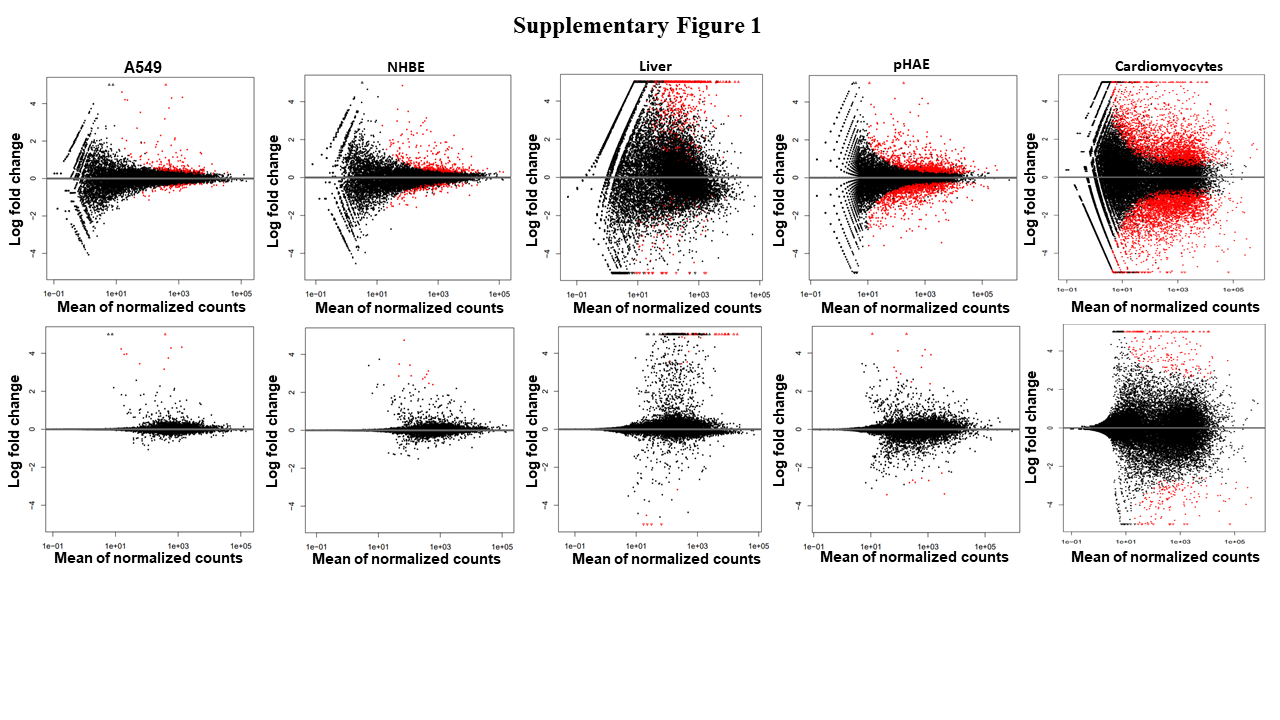


**Figure S1: SARS-CoV-2 mediated differentially expressed genes (DEGs) in various cells (Related to Fig 1).** Upper panel showing initial DEGs upon SARS-CoV-2 infection. Lower panel showing DEGs after applying `apelgm` function in DESeq2.


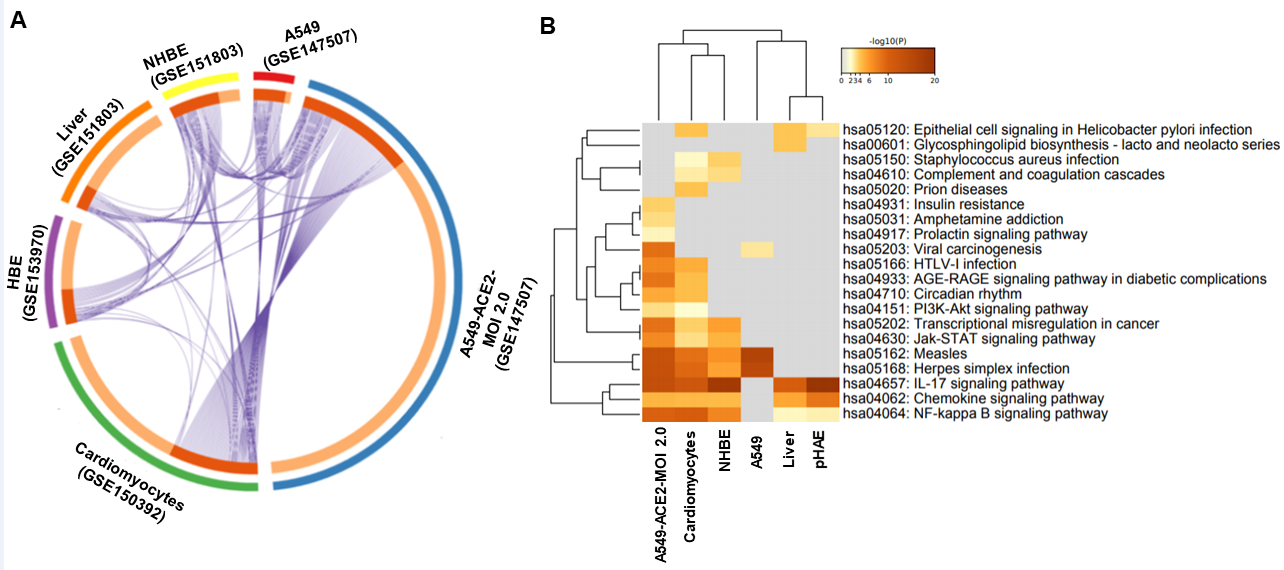


**Figure S2: Exogenous expression of ACE2 induced the number of DEGs upon SARS-CoV-2 infection (Related to Fig 2).** (A) Circos plot showing overlapping of significantly upregulated genes following SARS-CoV-2 infection using purple lines. (B) Hierarchical clustering of the top most enriched KEGG pathways from different datasets. Dendogram is colored by the p values where grey cells indicate the lack of significant enrichment.


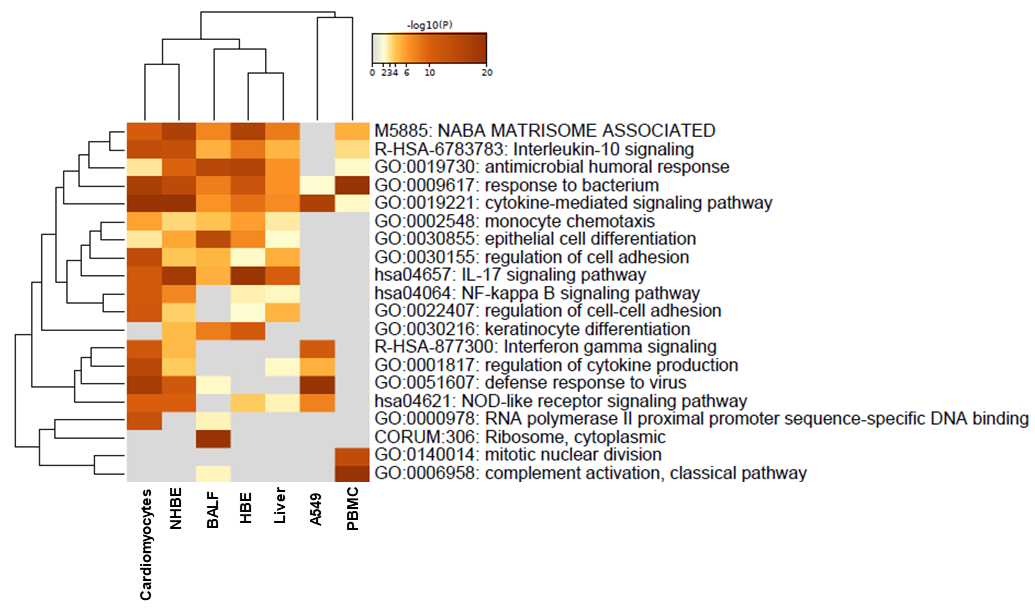


**Figure S3: Clinical data obtained from BALF and PBMC of COVID-19 patients was clustered with cellular infection data (Related to Fig 2).** Hierarchical clustering of the top most enriched KEGG pathways from different datasets. Dendogram is colored by the p values where grey cells indicate the lack of significant enrichment.
